# Laboratory evaluation of a new integrative assay to phenotype plasma fibrinolytic system

**DOI:** 10.1186/s12959-022-00435-6

**Published:** 2022-12-05

**Authors:** Marion Bareille, Michael Hardy, Bernard Chatelain, Thomas Lecompte, François Mullier

**Affiliations:** 1 Université Catholique de Louvain, CHU UCL Namur, Namur Thrombosis and Hemostasis Center (NTHC), Namur Research Institute for Life Sciences (NARILIS), Hematology Laboratory, Yvoir, Belgium; 2 Université Catholique de Louvain, CHU UCL Namur, Namur Thrombosis and Hemostasis Center (NTHC), Namur Research Institute for Life Sciences (NARILIS), Department of Anesthesiology, Yvoir, Belgium; 3 Université de Lorraine, Nancy, France; 4 Université de Namur, Department of Pharmacy, Namur Thrombosis and Hemostasis Center (NTHC), Namur Research Institute for Life Sciences (NARILIS), Namur, Belgium

**Keywords:** Euglobulin clot lysis time, Fibrinolysis, Global fibrinolytic capacity, Lysis Timer, Plasminogen activator inhibitor-1

## Abstract

**Background:**

There is currently no universal and standardized test available to phenotype plasma fibrinolytic system.

**Aims:**

Our main aims were to evaluate the performances of the ‘global fibrinolysis capacity’ assay (GFC) performed with the Lysis Timer® instrument, and to study the influence of some preanalytical conditions.

**Method:**

Euglobulin clot lysis time (ECLT) and GFC were performed under several preanalytical conditions.

**Results:**

GFC showed satisfactory intra- and inter-run precision. Frozen controls and reagents showed stability over the studied period.

There was no statistically significant difference between GFC assessed in plasma samples processed at 4 °C or at 20 °C. GFC assessed with frozen-thawed plasma samples was prolonged when compared to fresh samples (*p* = 0.014). The centrifugation scheme had no influence on PAI-1 activity levels, GFC and ECLT.

Reference interval for GFC ranges from 29.3 (C I90% = 26.9–31.9) to 49.5 (90% CI = 45.9–52.2) minutes.

In addition, a preliminary study in 40 healthy volunteers and 43 adult patients referred for investigation of a bleeding disorder was conducted to compare GFC and ECLT assays in their ability to classify samples with shortened or prolonged clot lysis times. Disagreements between ECLT and GFC were observed for 23 samples (out of 83), most of them minor.

**Conclusion:**

GFC is suitable and convenient for a broad clinical use and can be performed with frozen-thawed plasma samples.

Unlike ECLT, GFC is designed to take into account the balance between inhibitors and activators of the fibrinolytic system and could detect both hypo- and hyperfibrinolytic states. Whether it is as suitable as or even better than ECLT to detect a bleeding tendency due to a hyperactive fibrinolytic system deserves to be properly investigated.

## Introduction

The fibrinolytic system is involved in many physiological and pathological processes. An increase in fibrinolytic activity favours a bleeding tendency, whereas a reduced activity can contribute to the pathogenesis of thromboembolic diseases [1]. The assessment of fibrinolysis and its components seems therefore clinically relevant in the investigation of haemostatic disorders even though there is a lack of guidance for the laboratory assessment of hyperactive fibrinolytic system.

Fibrinolysis is tightly regulated by several inhibitors such as alpha-2-antiplasmin, thrombin-activatable fibrinolysis inhibitor (TAFI), hydroxyproline-rich glycoprotein (HRGP) and plasminogen activator inhibitor-1 (PAI-1). The latter belongs to the serine protease inhibitor family (as known as “serpin”), is the main inhibitor of both tissue-type (t-PA) and urokinase-type (u-PA) plasminogen activator and exists in plasma into three forms: a free one known as “active PAI-1”, an inactive one mainly complexed with t-PA or u-PA, and a latent form, resulting from a conformational change in the active PAI-1 molecule, called “latent PAI-1” [2].

Several laboratory methods exist to evaluate the fibrinolytic system, but no universal and standardized in vitro assay is currently available [3]. Assays can be divided into two categories: individual determination of the components of the fibrinolytic system, and coagulolytic tests assessing the fibrinolysis process as a whole in a clotting milieu [1]. Both approaches have strengths and limitations: coagulolytic assays are often used as screening tools to explore fibrinolysis; individual determination of the components of fibrinolysis is often restricted to its main components (PAI-1 and alpha-2-antiplasmin in case of bleeding tendency, PAI-1, plasminogen and t-PA in case of recurrent idiopathic thrombosis) and does not reflect their interactions, leading to the risk of missing a clinically relevant anomaly. Viscoelastic clot formation and lysis assays have also been described as potential useful tools to investigate fibrinolysis, with three comercially available assays: the TPA-test provided with the ClotPro® device, the APTEM assay provided with the ROTEM® device and the Qstat cartridge provided with the Quantra® device. Home-made assays such as the TEM-tPA have also been described [4–6].

Furthermore, as fibrinolysis is a very slow physiological process because of low or even no circulating levels of free plasminogen activators (fully complexed to PAI-1 and thus inactive) [7], two laboratory tips have to be used to speed up the process, leading to two different kinds of coagulolytic assays: on the one hand removal of most inhibitors (i.e. euglobulin clot lysis time, which relies on a very binding and time-consuming processing of plasma), and on the other hand addition of a low quantity of t-PA (i.e. global fibrinolytic capacity of plasma). Both approaches have pros and cons: the first modifies the baseline equilibrium and does not reflect physiological conditions, the second is sensitive to the amount of t-PA added, which should be sufficient to shorten lysis time but low enough not to alter the balance between all the actors of the fibrinolytic system [7, 8].

Euglobulin clot lysis time (ECLT), also known as von Kaulla assay, is a coagulolytic assay historically thought to detect what is still usually referred to as hyperfibrinolysis. This assay is commonly performed in our laboratory as part of the diagnostic workup for bleeding disorders, but suffers from four major limitations: (i) it is time-consuming, (ii) samples have to be drawn and processed on melting ice [9, 10], (iii) there is no harmonized and standardized procedure between laboratories, (iv) and as it is performed with a plasma sample artificially depleted in fibrinolysis inhibitors (mostly alpha-2-antiplasmin with less than 10% left, and PAI-1 and TAFI with respectively 42% and 38% left [11]); it does not reflect the whole in vivo phenomenon. To minimize inter-operator variability about the determination of the result, our center uses a photometer device, the Mevatronic T800 (MevaTronics, Belgium) [12], to record turbidity changes and to determine the clot lysis time.

Recently, a new device has been launched, the Lysis Timer® (HYPHEN BioMed, Neuville sur Oise, France), enabling the assessment of the so-called ‘global fibrinolysis capacity’ of plasma (GFC) with a short turn-around time (TAT) compatible with emergency settings and according to a standardized protocol [13]. Although this assay is of special interest for analyzing hypofibrinolysis due do its rationale (PAI-1 uses to be in excess in disease states, which induces the fibrinolytic potential for a defined amount of released t-PA), it showed a good sensitivity to hyperfibrinolysis in the liver transplantation context [14, 15], but a variable one in the trauma field [16]. However, it has not yet been evaluated in patients with known history of bleeding, and preliminary data are scarce concerning preanalytical issues [13] particularly with regard to the influence of the residual platelet count and that of a freeze-thawing cycle. Indeed, platelet α-granules have been reported as a circulating pool of PAI-1, both in its active and inactive form [17–19]. Since a freeze-thawing cycle disrupts platelet membranes leading to the release of platelet constituents such as PAI-1, and as ECLT and GFC are both sensitive to PAI-1 plasma levels [13, 20], we hypothesized that residual platelets in plasma could be a source of PAI-1 that might influence GFC and ECLT results.

The new regulation on in vitro diagnostic medical devices (IVDR EU 2017/746) implies that clinical performance of an assay (that is to say the ability of this assay to yield results correlated with a particular clinical condition or a physiological or pathological process or state in accordance with the target population and intended user) should be demonstrate before its implementation in daily practice [21]. We therefore conducted a thorough study of the GFC assay with the following aims:

i) to evaluate the performances (intra- and inter-run assay precision, stability of frozen controls and reagents) of the GFC assay;
ii) to study the influence of processing temperature (4 °C versus 20 °C);
iii) to study the influence of freeze-thawing cycles;
iv) to study the influence of centrifugation scheme on residual platelet count, PAI-1 level, GFC and ECLT results;
v) to assess reference intervals with fresh plasma samples from healthy subjects.

In addition, 40 healthy volunteers and 43 adult patients referred for investigation of a bleeding disorder were enrolled to compare GFC and ECLT assays in their ability to classify samples with shortened or prolonged clot lysis times.

## Materiel and methods

The study was approved by the local Ethics Committee (NUB: B0392020000042).

Blood samples from patients and healthy volunteers were collected from November 2019 until January 2022.

### Study population

Forty-three adult patients referred to the laboratory of our institution for investigation of a bleeding disorder based on clinical symptoms and a suggestive bleeding score were enrolled. The bleeding assessment tool used in our institution is the condensed MCMDM-1 VWD one [22].

Forty apparently healthy volunteers were recruited among the employees of our institution. Exclusion criteria were anemia, thrombocytopenia, abnormal prothrombin time (PT) or activated partial thromboplastin time (aPTT), abnormal fibrinogen level, known history of a coagulation disorder, recent intake of drugs affecting hemostasis, recent infection, ongoing pregnancy and post-partum < 3 months, and strenuous physical activity during the last 24 h.

For practical reasons, the influence of processing temperature (4 °C versus 20 °C) and that of a freeze-thawing cycle were assessed on respectively 30 (20 healthy volunteers, 10 patients) and 40 (30 healthy volunteers, 10 patients) subjects; the influence of centrifugation scheme was studied on 10 healthy male volunteers; and reference interval was established with 22 (11 women, 11 men) healthy volunteers.

### Blood collection and processing

Blood samples from fasting patients and healthy volunteers were drawn between 8:30 and 10:30 a.m. after at least 20 min of rest by venipuncture and collected into 109 mM buffered citrated, dried tubes, and K2-ethylenediaminetetraacetic acid (EDTA) dried sprayed tubes (BD Vacutainer®, Becton Dickinson, Erembodegem, Belgium) using a 21-gauge needle. The tourniquet pressure was released during the filling of the discard tube. Blood samples were collected according to the following order: discard tube, sodium citrated tubes, dried tubes, and K2-EDTA dried sprayed tube. Each tube was inverted once immediately after collection and before collecting the next tube, and inverted four more times at the end of the collection according to EFLM-COLABIOCLI guidelines [23]. Sodium citrate containing tubes dedicated to the ECLT assay and to study the influence of the processing temperature on GFC were kept on melting ice from the sampling time whereas the others remained at room temperature (20 °C).

Citrated platelet-depleted plasma (c-PDP) was prepared according to the recommendations of the International Council for Standardization in Haematology (ICSH) [24] and as per daily practice. Citrated blood samples were centrifuged at 1,500 g for 15 min (Mega Star 3.0R, VWR International, Leuven, Belgium – breaking ramp 9) either at 4 or 20 according to the performed assay and protocol (see below) within 2 h of blood collection. The upper two thirds of supernatants were collected and underwent a second centrifugation at 1,500 g for 15 min at either 4 or 20 °C. Double centrifuged supernatants, i.e. c-PDP, were either analyzed within 1 h or stored at -80 °C (500–800 µL—Free standing cryogenic vials 2 mL PP, VWR International, Leuven, °C Belgium) until further analysis. In that latter case, frozen plasma samples were thawed 5 min at 37 °C and kept 15 min at room temperature before analysis as per daily practice.

### Laboratory methods

Hemoglobin level and platelet count were assessed in K2-EDTA anticoagulated whole blood samples by sodium lauryl sulphate (SLS) hemoglobin detection method and impedance-based counting method by a Sysmex XN-20 analyzer (Sysmex Corporation, Kobe, Japan).

Prothrombin time (estimated by the prothrombin ratio with results expressed as a percentage by extrapolation of the subject's measured PT using the Thivolle regression line), aPTT and fibrinogen level were measured in fresh, citrated, double-centrifuged plasma samples, with a STA-R Max 2 analyzer using the following reagents: STA-NeoPTimal (Stago, Asnières-sur-Seine, France) for the PT, CKPrest (Stago, Asnières-sur-Seine, France) for the aPTT, and STA-Liquid FIB (Stago, Asnières-sur-Seine, France) for the Clauss fibrinogen.

Euglobulin clot lysis time (ECLT) was assessed with fresh and frozen c-PDP samples using the Mevatronic T800 (MevaTronics, Belgium) [12]. Briefly, 500 µL of c-PDP was diluted 1:20 with acetic acid 0.014%, incubated 15 min at 4 °C and centrifuged 15 min at 1,500 g at °C. Supernatant was discarded and the pellet was dissolved in 500 µL of Owren-Koller buffer (Stago, Asnières-sur-Seine, France). 400 µL were then transferred to a cuvette and the cuvette was introduced into the Mevatronic T800. Clot formation was triggered by 100 µL of CaCl_2_ 0.025 M (Stago, Asnières-sur-Seine, France) and light transmittance was continuously recorded at a wavelength of 890 nm. The digital signal was then converted into a light transmission curve and its primary and secondary derivatives by the dedicated software [12]. Locally established reference interval ranges from 131 to 523 min.

Global fibrinolysis capacity of the plasma (GFC) was assessed in citrated double-centrifuged fresh and frozen plasma samples using the new generation of the Lysis Timer ® (HYPHEN BioMed, Neuville sur Oise, France) with the dedicated reagents (Hyphen Biomed, Neuville-sur-Oise, France) as previously described [13]. To make it brief, 100µL of c-PDP was mixed with 100 µL of reagent R1 containing recombinant t-PA (100 ng/mL) and silica, and incubated at 37 °C in a cuvette introduce into one of the wells of the Lysis Timer® device. Clot formation was then triggered by 100 µL of reagent R2 containing human thrombin (4 NIH/mL) and CaCl_2_ (concentration not provided by the manufacturer). Light transmittance was continuously measured at a wavelength of 940 nm and the digital signal was then converted into a light transmission curve and its primary and secondary derivatives by the dedicated software.

Intra- and inter-run precisions for GFC were assessed using the three-level lyophilized quality controls provided by Hyphen Biomed. Controls and reagents were reconstituted and kept no more than 7 days at 4 °C as recommended by the manufacturer. For inter-run precision, measurements were performed twice daily during the first ten days, then once a day from day 11 to day 25 resulting in 35 measures. Lyophilized quality controls provided consists of pooled normal plasma ‘native’ (level 2) or spiked either with t-PA (level 1) to mimic hyperfibrinolysis or with aprotinin (level 3) to mimic hypofibrinolysis.

Stability of frozen controls and reagents were studied over a period of sixty-five and forty-three days respectively. Controls and reagents from the same batch were reconstituted, pooled, aliquoted (150 µL for controls – Multiply-Pro cup 0.5 mL PP, Sarstedt, Nümbrecht, Germany; 300 µL for reagents—Safe-lock tubes 1.5 mL, Eppendorf, Aarschot, Belgium) and stored at -80 °C until analysis. A normal c-PDP sample was obtained from a healthy volunteer, aliquoted (500 µL—Free standing cryogenic vials 2 mL, VWR International, Leuven, Belgium) and frozen at -80 °C until analysis. Frozen controls were thawed 5 min at 37 °C and kept 15 min at room temperature before analysis with reconstituted reagents kept no more than 7 days at 4 °C. Frozen reagents and normal plasma were thawed 5 min at 37 °C and kept 15 min at room temperature before analysis.

**Fig. 1:**
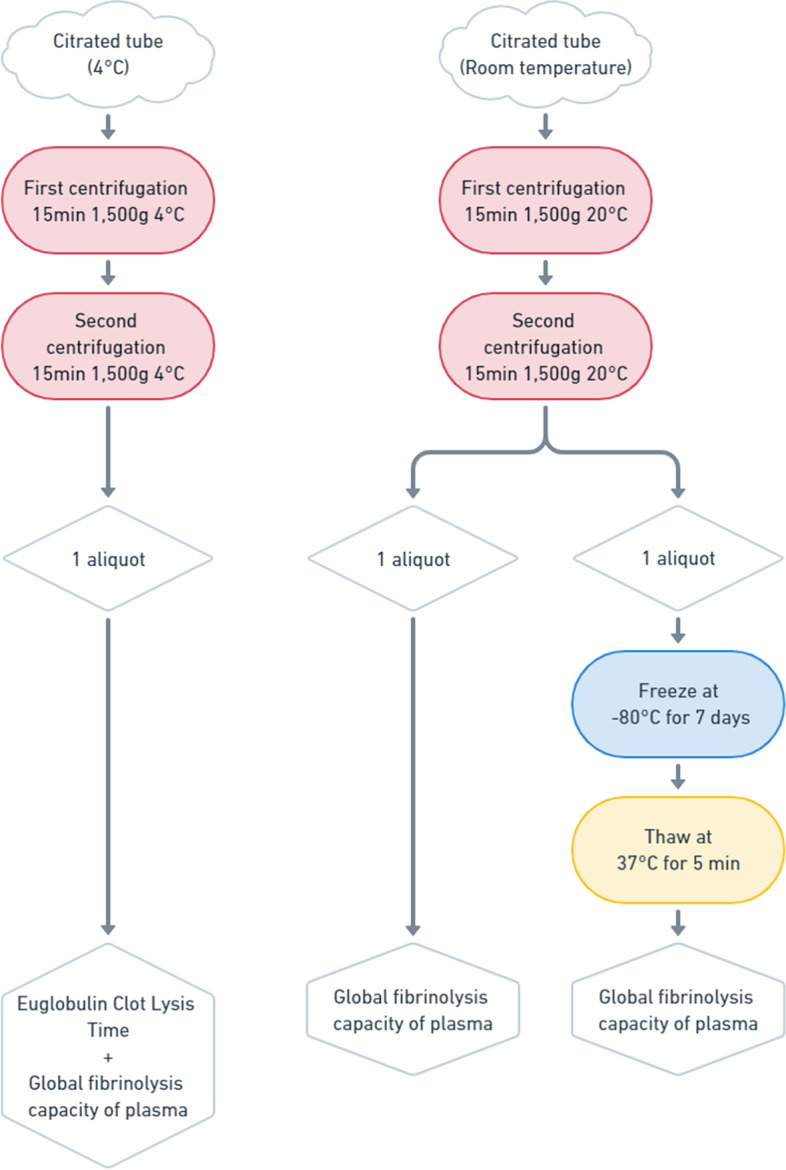
Experimental design for the influence of processing temperature and a freeze-thawing cycle

The influence of processing temperature (4 °C versus 20 °C) and that of a freeze-thawing cycle on GFC were described in Fig. 1. Briefly, from each subject’s blood sample three plasma aliquots were processed: one from the citrate-containing tube kept on melting ice and processed at 4 °C for the ECLT assay, which was analyzed right after the second centrifugation, and two from a citrate-containing tube processed at room temperature. Among them, one was analyzed right after the second centrifugation and the other (500 µL—Free standing cryogenic vials 2 mL, VWR International, Leuven, Belgium) was stored at -80 °C for 7 days before thawing 5 min at 37 °C and analysis.

**Fig. 2:**
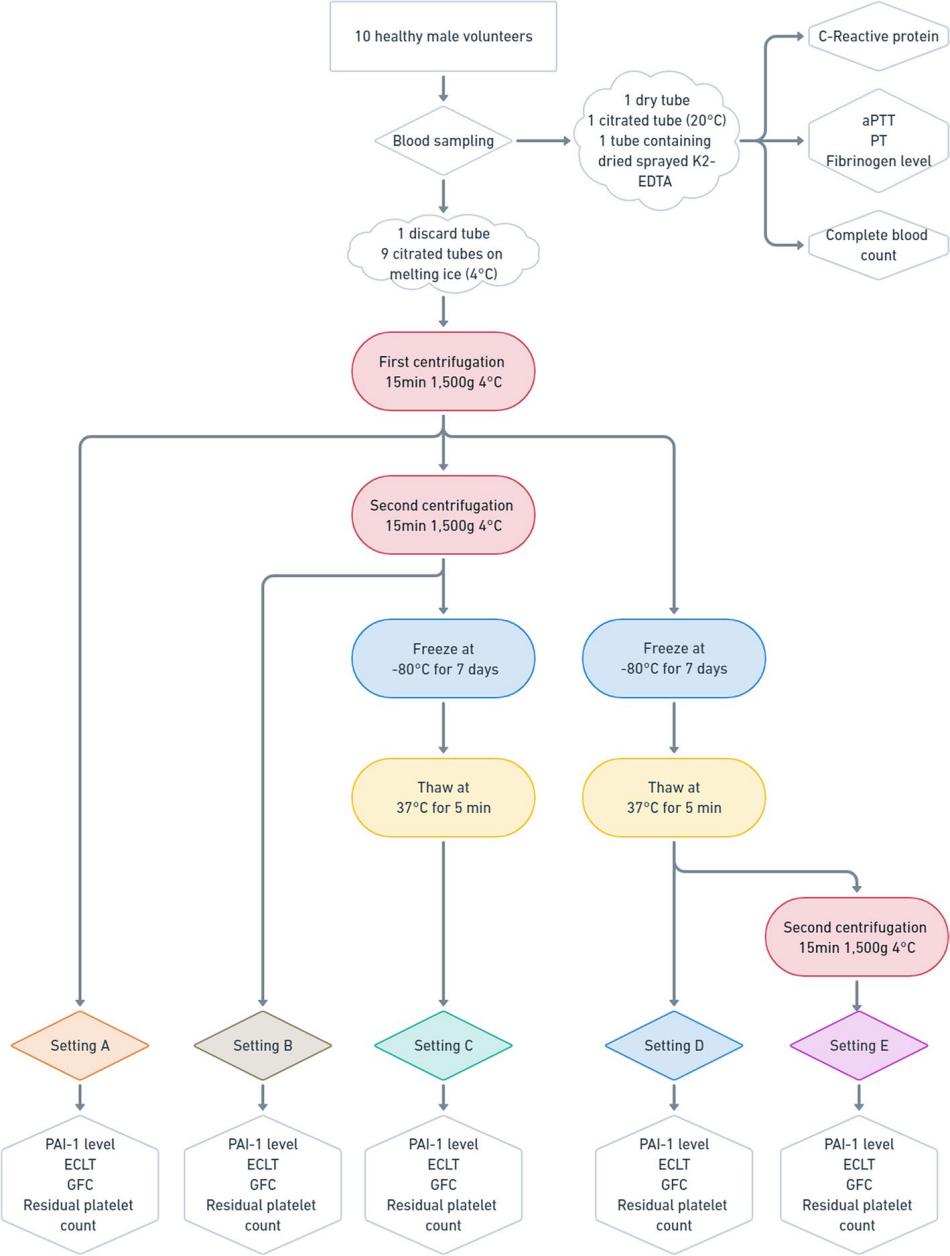
Experimental design for the influence of centrifugation scheme. Settings: (A) single centrifugation (1,500 g, 15 min) with no freeze-thawing cycle before analysis, (B) double centrifugation (1,500 g, 15 min) with no freeze-thawing cycle before analysis, (C) double centrifugation (1,500 g, 15 min) before a freeze-thawing cycle and analysis, (D) single centrifugation (1,500 g, 15 min) before a freeze-thawing cycle and analysis, (E) one centrifugation (1,500 g, 15 min) before and another (1,500 g, 15 min) after a freeze-thawing cycle before analysis

The influence of centrifugation parameters on residual platelet count, PAI-1 level, and GFC and ECLT results was assessed with c-PDP samples from blood collected from 10 healthy male volunteers prepared under five different pre-analytical conditions (Settings A to E) as described in Fig. 2:

(A) single centrifugation (1,500 g, 15 min) with no freeze-thawing cycle before analysis,
(B) double centrifugation (1,500 g, 15 min) with no freeze-thawing cycle before analysis,
(C) double centrifugation (1,500 g, 15 min) before a freeze-thawing cycle and analysis,
(D) single centrifugation (1,500 g, 15 min) before a freeze-thawing cycle and analysis,
(E) one centrifugation (1,500 g, 15 min) before and another (1,500 g, 15 min) after a freeze-thawing cycle before analysis.

As PAI-1 is present in plasma in three forms (active, inactive, and latent) we have decided to use a functional assay to measure PAI-1 activity rather than an immunoassay to determine PAI-1 antigen. PAI-1 activity level was then measured with a STA-R Max 2 analyzer using Stachrom PAI-1 reagent (Stago, Asnières-sur-Seine, France). Residual platelet count was assessed with a fluorescence method (PLT-F channel) by a Sysmex XN-20 analyzer (Sysmex Corporation, Kobe, Japan).

Reference interval was determined from 22 healthy volunteers’fresh c-PDP samples.

Method comparison between ECLT and GFC was performed with 83 residual anonymized citrated frozen plasma samples collected either from patients with personal history of bleedings (*n* = 43) or from healthy adult volunteers (*n* = 40).

### Statistical analysis

Statistical analysis was performed using MedCalc Statistical Software version 14.8.1 (MedCalc Software bvba, Ostend, Belgium) for the assessment of the reference interval according to CLSI C28-A3 robust method, and R Statistical Software (v4.1.2; R Core Team 2021) through RStudio (RStudio, PBC, Boston, MA, USA) and the following packages: dplyr [25], tidyverse [26], ggstatsplot [27] and ggplot2 [28].

Data are expressed as mean ± standard deviation (SD) for normally distributed variables or as median [interquartile range [IQR (25–75)]] for the others. Normality was assessed by analyzing the histogram distribution and the normal Q-Q Plot, and by Shapiro–Wilk test for each variable. Quantitative data were compared using Student t-tests or non-parametric signed rank tests (Wilcoxon or Mann–Whitney test), as appropriate. To assess the influence of the processing temperature or of a freeze-thawing cycle, we also used the Krouwer method derived from Bland and Altman method) as we plotted differences between the two methods (e.g. GFC after and GFC before a freeze-thawing cycle) against one of the two methods considered as the reference (e.g. GFC before a freeze-thawing cycle). Changes in laboratory parameters according to the centrifugation scheme were studied with ANOVA for repeated measures or with a Friedman test as appropriate. A *p*-value < 0.05 or adjusted with Bonferroni correction in the event of multiple comparison tests was considered statistically significant. Correlation between both assays and between PAI-1 plasma levels and GFC and ECLT was evaluated by Pearson or Spearman correlation coefficient as appropriate.

Reference range interval was determined from 22 healthy volunteers’fresh plasma samples using robust method, according to CLSI C28-A3 guidance [29].

## Results

### Intra and inter-run precision

**Fig. 3:**
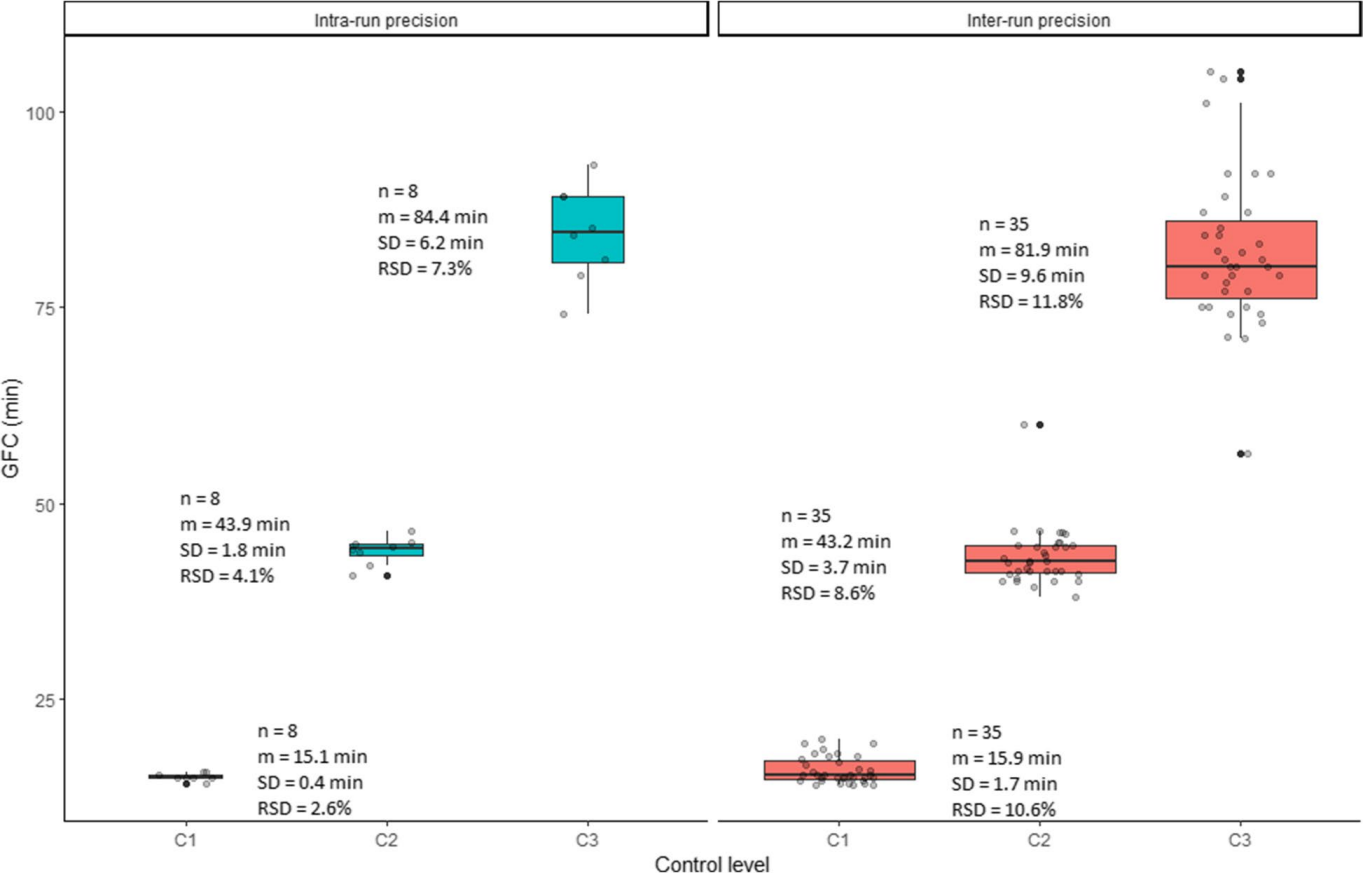
Summary of intra and inter-run precision for GFC. The line dividing the box into two parts represents the median of the data. The ends of the box show the upper (Q3) and lower (Q1) quartiles. The extreme line shows the highest and lowest value excluding potential outliers according to the following formula: Q3 + 1.5xIQR (maximum) and Q1-1.5xIQR (minimum). Black dots beyond the extreme line show potential outliers, grey dots represent individual data. Mean (m), standard deviation (SD) and relative standard deviation (RSV) are given as supplementary indicators

‘Global fibrinolysis capacity’ of the plasma showed a satisfactory intra- and inter-run precision, with a standard deviation and a relative standard deviation of respectively 0.4 min and 2.6% (level 1), 1.8 min and 4.1% (level 2), and 6.2 min and 7.3% (level 3) for intra-run precision; and of 1.7 min and 10.6% (level 1), 3.7 min and 8.6% (level 2), and 9.6 min and 11.8% for inter-run precision (Fig. 3).

### Stability of frozen controls and reagents

**Fig. 4:**
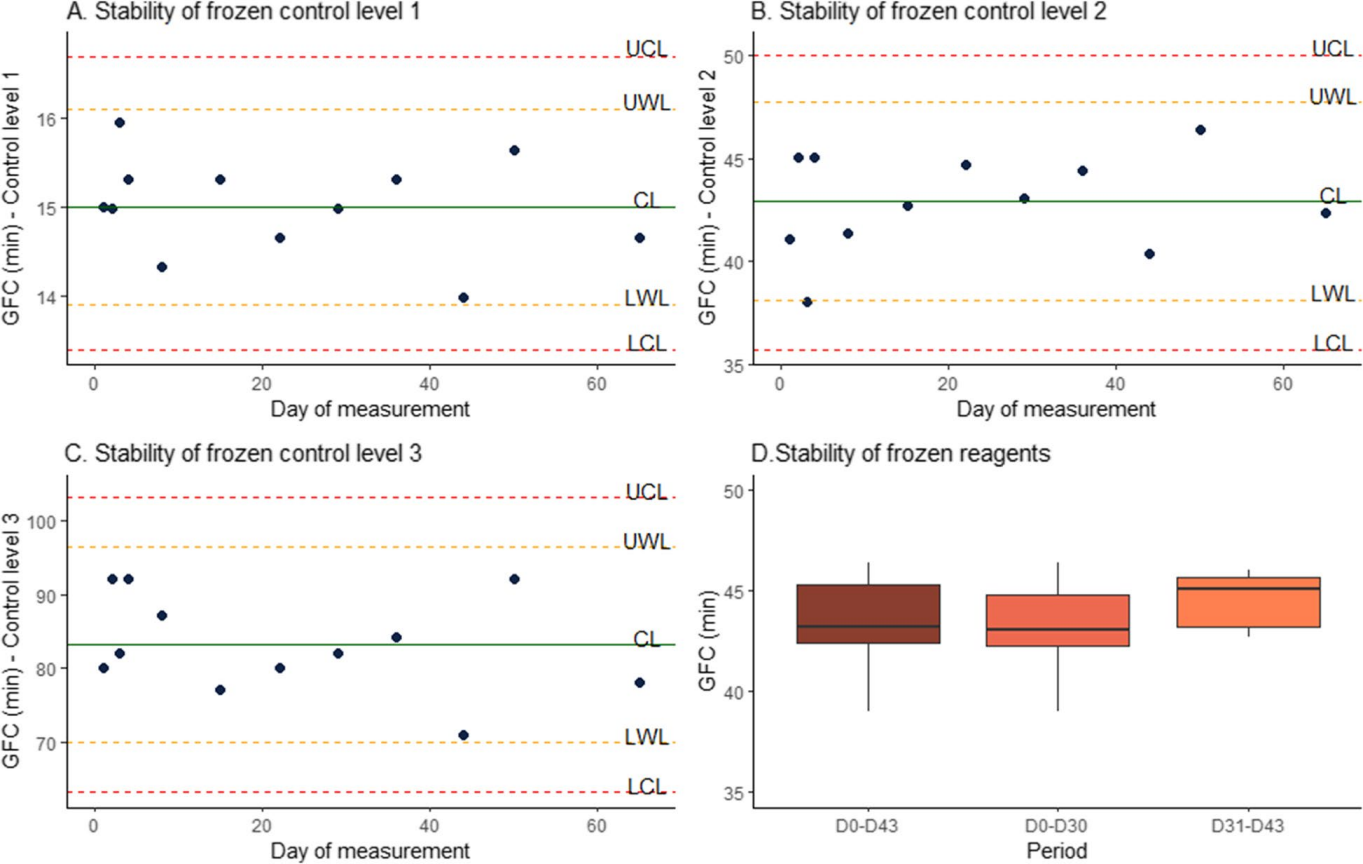
Stability of controls (**A**, **B**, **C**) and reagents (**D**) kept aliquoted and frozen. UCL: Upper control limit (m + 3 SD); UWL: Upper warning limit (m + 2 SD); CL: Central line (m); LWL: Lower warning limit (m – 2 SD); LCL: Lower control limit (m + 3 SD). D0-D43 represents the whole 43 days-period; D0-D30 represents the first 30 days (given as the limit by the manufacturer); and D31-D43 represents the last 13 days (extended period)

All of the three levels of frozen controls showed stability over the studied 65-day period (60 days according to the manufacturer), with all values belonging to the m ± 2 s interval (orange lines) for control levels 1 and 3, and with all values belonging to the m ± 3 s interval (red lines) and 11/12 values belonging to the m ± 2 s interval (orange lines) for control level 2 (Fig. 4 A, B and C). Standard deviation and relative standard deviation were respectively of 0.6 min and 3.7% (level 1), 2.4 min and 5.6% (level 2), and 6.6 min and 8% (level 3).

Frozen reagents showed stability over a 43 days period (30 days according to the manufacturer), with a standard deviation and a relative standard deviation of respectively 2 min and 4.5% (Fig. 4 D). Mean and standard deviation did not differ between the first 30 day-period (day 1 to 30) and the last 13 day-period (day 31 to 43) (*p* = 0.166 and *p* = 0.545).

### Influence of the processing temperature

**Fig. 5:**
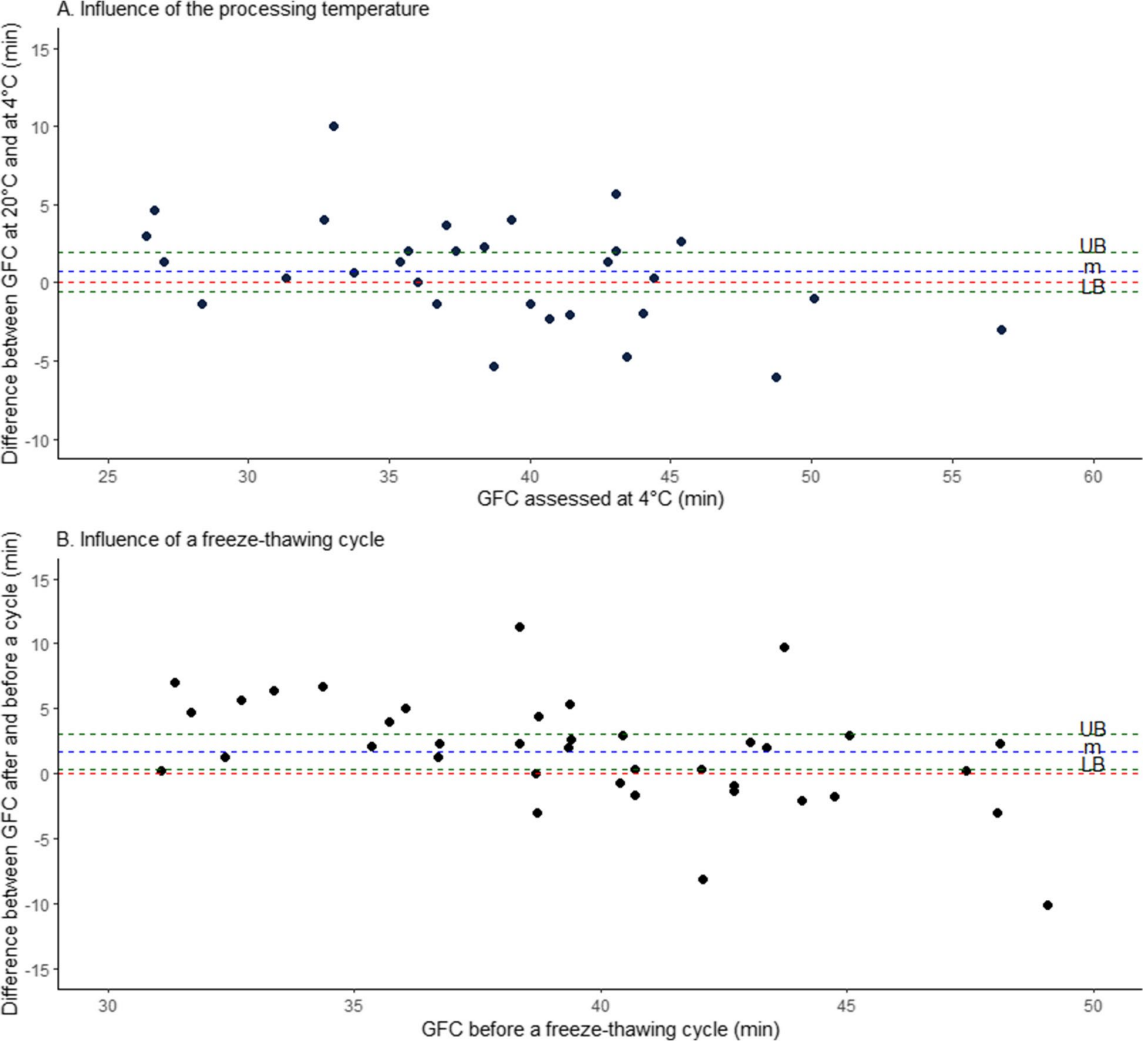
Influence of the processing temperature (**A**) and a freeze-thawing cycle (**B**) on GFC. The red dashed line would indicate the absence of difference between the two settings. The blue dashed line represents the mean (m) of the differences between the two settings and the green ones represent the lower (LB) and the upper (UB) bound of 95% confidence interval for mean of the differences. **A** Influence of the processing temperature on GFC: GFC assessed at 4 °C was considered as the reference (by analogy with ECLT). Differences between GFC assessed at 20 °C and at 4 °C for each subject were plotted against GFC assessed at 4 °C. **B** Influence of a freeze-thawing cycle on GFC: GFC assessed with a fresh sample before a freeze-thawing cycle was considered as the reference. Differences between GFC assessed after and before a freeze-thawing cycle for each subject were plotted against GFC before a freeze-thawing sample

There was no statistically significant difference between GFC assessed in plasma samples kept and processed on melting ice (4 °C) or at room temperature (20 °C) (*p* = 0.273), with a mean of the differences (blue dashed line) of 0.7 min (95% CI = -0.58–1.98, green dashed lines) (Fig. 5 A).

### Influence of a freeze-thawing cycle

GFC assessed in frozen-thawed plasma samples is prolonged when compared to fresh samples (*p* = 0.014), with a mean of the differences (blue dashed line) of 1.74 min (95% CI = 0.37–3.12, green dashed lines) (Fig. 5 B).

### Influence of centrifugation and residual platelet count on plasma PAI-1 activity levels, euglobulin clot lysis time (ECLT) and global fibrinolytic capacity (GFC) of plasma

Correlation of plasma PAI-1 activity levels with ECLT and GFC was statistically highly significant, with a Pearson coefficient of 0.63 (*p* < 0.0001) and 0.64 (*p* < 0.0001), respectively.

**Fig. 6:**
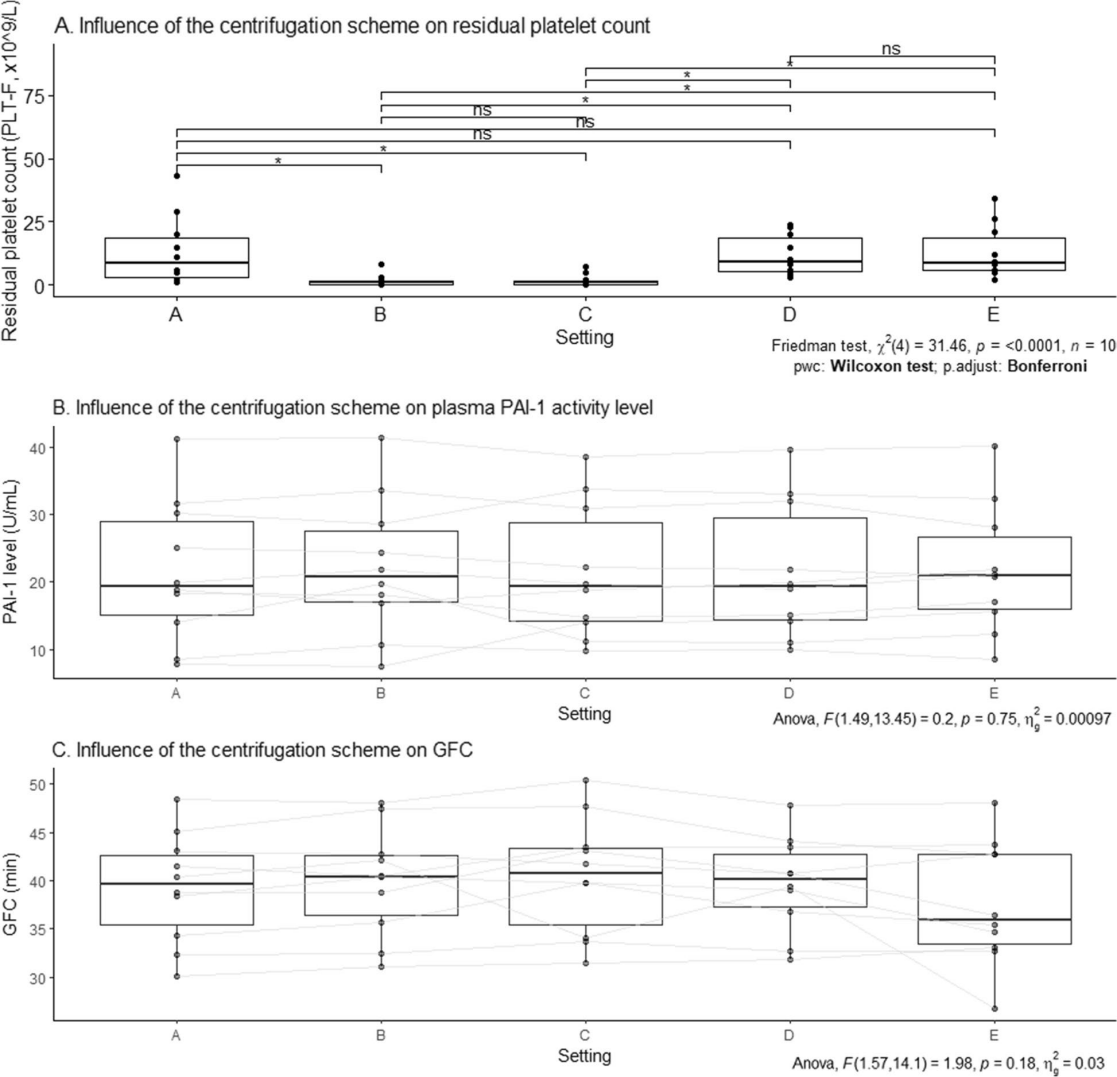
Influence of the centrifugation scheme on residual platelet count (**A**), PAI-1 activity level (**B**) and GFC (**C**). Settings: (A) single centrifugation (1,500 g, 15 min) with no freeze-thawing cycle before analysis, (B) double centrifugation (1,500 g, 15 min) with no freeze-thawing cycle before analysis, (C) double centrifugation (1,500 g, 15 min) before a freeze-thawing cycle and analysis, (D) single centrifugation (1,500 g, 15 min) before a freeze-thawing cycle and analysis, (E) one centrifugation (1,500 g, 15 min) before and another (1,500 g, 15 min) after a freeze-thawing cycle before analysis. The line dividing the box into two parts represents the median of the data. The ends of the box show the upper (Q3) and lower (Q1) quartiles. The extreme line shows the highest and lowest value excluding potential outliers according to the following formula: Q3 + 1.5xIQR (maximum) and Q1-1.5xIQR (minimum). Black dots beyond the extreme line show potential outliers, grey dots represent individual data. *p < 0.05; ns = not significant

Residual platelet counts differed according to the centrifugation scheme (Fig. 6 A, *p* < 0.0001) as expected, but PAI-1 activity levels did not (Fig. 6 B, *p* = 0.75) nor GFC (Fig. 6 C, *p* = 0.18) and ECLT (*p* = 0.091).

### Reference interval

Hemoglobin levels (*p* < 0.001), platelet counts (*p* = 0.043), and ECLT (*p* = 0.003) differed according to the gender (Table 1).

**Table 1:** Healthy volunteers’ laboratory characteristics

Variables	Female (*n* = 11)	Male (*n* = 11)	*P*-value	Total (*n* = 22)
Age (y)	34 [26.8 – 44]	33 [28.5 – 47.8]	0.782	33.5 [28–45]
Haemoglobin (g/dL)	13.6 ± 0.7	15.1 ± 0.8	**0.0001**	14.3 ± 1.1
Platelet count (× 10^9^/L)	288 ± 47	239 ± 59	**0.043**	263 ± 58
Prothrombin Time (%)	100 [97 – 100]	96 [93—100]	0.178	98 [94—100]
Activated Partial Thromboplastin Time (sec)	30.7 ± 2.1	30.2 ± 1.3	0.513	30.4 ± 1.7
Fibrinogen (mg/dL)	312 ± 57	291 ± 30	0.288	301 ± 46
Euglobulin Clot Lysis Time (min)	249.3 ± 59	390.3 ± 120.1	**0.003**	319.8 ± 117.2
Global Fibrinolysis Capacity (min)	39.6 ± 5.1	39.5 ± 4.5	0.943	39.5 ± 4.7

Data are presented as mean ± SD or as median and [interquartile range]

Prothrombin time is expressed as a percentage by interpolation of subject's measured prothrombin time using Thivolle regression line

According to CLSI C28-A3 robust method recommended for small sample sizes [29], GFC reference interval ranges from 29.3 (90% CI = 26.9–31.9) to 49.5 (90% CI = 45.9–52.2) minutes.

### Agreement of the assays for the classification of extreme phenotypes (shortened or prolonged lysis times)

ECLT and GFC were weakly correlated with a Spearman’s coefficient of 0.228 (*p* < 0.05).

**Fig. 7:**
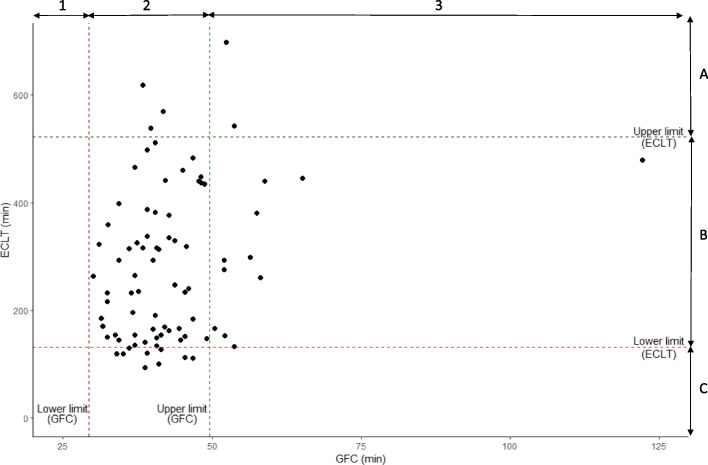
Distribution of samples according to the assay performed. The lower and upper limits used for ECLT and GFC correspond to the limits of locally established reference intervals. Samples located in zones A3, B2 and C1 show agreement between the lysis times provided by the two assays whereas samples located in zones A2, B3 and C2 show disagreements, most of them minor

Of 83 samples, nine showed a slightly shortened ECLT (< 131 min), but none had a decreased GFC (< 29.3 min) (Fig. 7, area C2). Thirteen displayed an increased GFC (> 49.5 min): two also had an increased ECLT (> 523 min, Fig. 7 area A3) whereas the 11 others had an ECLT within the locally established reference range (131—523 min, Fig. 7 area B3). Five displayed an increased ECLT (> 523 min): two also had an increased GFC (> 49.5 min, Fig. 7 area A3) whereas for the three others the GFC was within the locally established reference range (29.3 – 49.5 min, Fig. 7 area A2).

A sample showed a very prolonged GFC with an ECLT within the locally established reference range. Analysis was performed twice to exclude a technical issue. Due to a shortage of sample material, no additional analysis (PAI-1 activity, alpha-2-antiplasmin…) could be performed.

## Discussion

A high proportion of patients (up to 75%) referred for a mild bleeding tendency shows no lab abnormalities and are diagnosed with a bleeding disorder of undefined cause (BDUC) [30]. A recent survey focusing on current practice for diagnosis and management of patients with unclassified bleeding disorders in United Kingdom [31] showed that only 37% and 2% of the centres assayed PAI-1 and/or alpha-2-antiplasmin or performed euglobulin clot lysis time respectively to exclude other disorders before making a diagnosis of an unclassified bleeding disorder in a patient with a significant bleeding history. The laboratory work-up of a bleeding tendency would benefit a lot from a fibrinolysis assay sensitive enough to qualitative and/or quantitative defects in the fibrinolysis system, sufficiently robust to not be (too much) affected by preanalytical constraints (such as a freeze-thawing cycle or the sampling temperature), and standardised enough to provide comparable results among laboratories.

To our knowledge, as far as viscoelastometric tests are concerned, only the ClotPro® device is provided with a reagent (TPA-test) dedicated to the investigation of fibrinolysis, the APTEM (ROTEM®) and QStat (Quantra®) assays being rather oriented towards the detection of moderate to acute hyperfibrinolysis associated to trauma-induced coagulopathy (TIC) [32] than to a mild hyperfibrinolysis related to alpha-2-antiplasmin or PAI-1 deficiency [33]. Home-made assays such as TEM-tPA (a whole blood tissue factor activated ROTEM assay, with and without the addition of recombinant tissue plasminogen activator) suffer from a lack of standardisation, with a r-tPA concentration ranging from 50 to 625 ng/mL [4–6], and appear also to be more sensitive to the detection of hypofibrinolysis due to clot with reduced sensitivity to fibrinolysis rather than a mild hyperfibrinolysis related to alpha-2-antiplasmin or PAI-1 deficiency [5].

A few years ago, the Subcommittee on Factor XIII and Fibrinogen and the Subcommittee on Fibrinolysis of the ISTH published results on the feasibility of a standardized combined plasma clot turbidity and lysis assay [3]. They reported a significant interlaboratory variation (CVs of up to 50%) despite the use of the same protocol and lyophilized plasma from the National Institute for Biological Standards and Control (NIBSC), and they identified two critical factors contributing to this variation: timing (mixing and pipetting reagents, measurement of data points), and calculation of the different parameters from the data. The GFC estimation method using the Lysis Timer® device relies on protocol with reagents for commercial use, and is provided with a dedicated software to analyse clot lysis kinetics [13]. We therefore believe this assay could fulfil the recommendations of the ISTH Subcommittee, and could be a assay for the exploration of a fibrinolysis defect in a clinical laboratory.

The new regulation on in vitro diagnostic medical devices (IVDR EU 2017/746) implies that clinical performance of an assay (that is to say the ability of this assay to yield results correlated with a particular clinical condition or a physiological or pathological process or state in accordance with the target population and intended user) should be demonstrate before its implementation in daily practice [21].

GFC performance was consistent with the manufacturer's claims.

While the processing temperature did not have any significant influence on the GFC, a freeze-thawing cycle leads to a statistically significant increase in the GFC. Centrifugation scheme had no influence on plasma PAI-1 activity level, GFC or ECLT.

Finally, compared to ECLT there are fewer pre-analytical constraints since samples no longer need to be processed at 4 °C nor to be depleted in fibrinolysis inhibitors, leading to a shorter TAT compatible with emergency settings. However, we have observed disagreements between the two methods with samples with shortened or prolonged clot lysis times according to ECLT but within reference interval according to GFC, and conversely samples with prolonged clot lysis times according to GFC but within reference interval according to ECLT.

We discuss below those issues one by one.

### Analytical performance of the assay

Amiral et al. [13] have described relative standard deviation for intra- and inter-run precision less than 4% using frozen plasma samples prepared from collected blood of healthy volunteers. Our results (2.6, 4.1 and 7.3% for intra-run precision and 10.6, 8.6 and 11.8% for inter-run precision) were higher using the lyophilized quality controls provided, but similar to those obtained by Roullet et al. [14] for inter-run precision (13, 11 and 13%; intra-run precision was not evaluated).

Frozen aliquoted controls and reagents showed a very good stability over manufacturer’s recommended period and few days beyond (65 versus 60 days for controls, and 43 versus 30 days for reagents). It is likely to span a more extended period, but we have not measured it beyond days 43 and 65. This stability meets some laboratory’s needs concerning flexibility with the ability of performing analysis for non-urgent samples in batch with fresh reagents in one hand, and on the other hand to immediately analyze urgent samples using aliquoted frozen and thawed reagents.

### Influence of the processing temperature

To perform the euglobulin clot lysis time, samples have to be collected and processed on melting ice [9, 10] to preserve plasma t-PA and PAI-1 from degradation and to prevent spontaneous t-PA/PAI-1 complex formation. In contrast, the processing temperature has no influence on GFC, because plasma t-PA and active PAI-1 are stabilized and protected from early degradation or neutralization by interactions with all the other actors of the fibrinolytic system [2, 34].

GFC stability at room temperature has a positive impact: blood samples from patients no longer need to be collected and processed at 4 °C, leading to reduced preanalytical constraints compared to ECLT. Furthermore, collection and storage of citrated whole blood on ice prior to centrifugation could result in lower factor VIII and plasminogen levels [35, 36] that could potentially affect GFC results.

### Influence of a freeze-thawing cycle

A freeze-thawing cycle leads to a statistically significant increase in the GFC but the mean of the differences remains weak (1.74 min, 95% CI = 0.37–3.12) and close to intra and inter-run precision. Paradoxically, a freeze-thawing cycle has been described to substantially reduced PAI-1 activity [19] in plasma samples which should result in a shortening of the GFC and not a prolongation as observed here.

### Influence of centrifugation scheme

PAI-1, also known as Serpin E1, exists into three forms in plasma: free active PAI-1, inactive PAI-1 and latent PAI-1 [2]. Free active PAI-1 displays an unstable conformation leading to a short half-life of one hour once released into circulation. Binding to t-PA or vitronectin increases its half-life: PAI-1 complexed to t-PA is known as inactive PAI-1, whereas binding to vitronectin stabilizes PAI-1 into its active form and increases its half-life up to two to four hours [37]. Latent PAI-1 results of a loss of activity of active PAI-1 by spontaneous insertion of the reactive centre loop into the body of the molecule [38]: the inhibitory activity can be restored through denaturation and renaturation or by exposure to negatively charged phospholipids [34, 39, 40].

We have performed a functional assay to measure PAI-1 activity rather than an immunoassay aiming to determine PAI-1 antigen level. Although residual platelet counts substantially differed according to the centrifugation scheme, PAI-1 activity level, GFC and ECLT showed no difference according to the setting. These results are consistent with literature findings suggesting that platelet PAI-1 is significantly less active than plasma PAI-1 [17, 41] (with a median inhibitory activity towards t-PA of 1.6 units/mL for plasma PAI-1, versus 8.7 units/mL for platelet PAI-1 [17]), and that a freeze-thawing cycle has been described to substantially reduced PAI-1 activity [19] in plasma samples. Furthermore, a study showed that the median platelet contribution to PAI-1 activity was 0.017 units/10^6^ platelets, with a median specific activity of 26,000 units/mg, which was considerably lower than the median plasma PAI-1 specific activity of 116,000 units/mg [17]. PAI-1 released by residual platelets therefore seems an unlikely explanation for the GFC increase observed after a freeze-thawing cycle.

### Reference intervals

GFC reference interval ranges from 29.3 to 49.5 min with a median of 39.1 min and an IQR ranging from 36.4 to 43.7 min. Our reference interval was left-shifted compared to those determined by Amiral et al. (30–60 min) [13] and Roullet et al. (median = 44 min, with an IQR ranging from 41 to 49 min) [14]. These results argue for a local verification of the reference interval by each laboratory, as the indicative intervals provided by the manufacturer could not be fully adapted to the local population and settings [29].

Amiral et al. [13] have found that GFC was slightly higher in women than in men, with a progressive decrease with age consistent with an influence of a gender-related hormonal status. Within our population of healthy subjects, ECLT but not GFC differed according to gender, with a shorter ECLT in women than in man.

In a broader context, several tests are used to monitor clot lysis time: some of them display different results according to the gender, whereas for others there is no difference [20, 42]. This could be explained, at least in part, by a combination of two factors. First, the fibrinolytic system is tightly regulated and equilibrium results from a balance between inhibitors (such as PAI-1, α2-antiplasmin and α2-macroglobulin, and thrombin activatable fibrinolysis inhibitor (TAFI)) and activators (such as t-PA), with lower levels of plasma PAI-1, t-PA and t-PA/PAI-1 complexes in women before menopause [42–46].

Second, ECLT is predominantly sensitive to t-PA and residual plasma PAI-1 levels whose baseline values are lower in women, because of the removal of the majority of the fibrinolysis inhibitors. A study has shown that after processing of the plasma there were only 7%, 38% and 42% left of respectively α-2-antiplasmin, TAFI and PAI-1 antigen against 91% and 65% for t-PA and plasminogen [11]. In contrast, as GFC is performed on ‘native’ plasma samples taking in account all the actors of the fibrinolytic system, the assay should be less sensitive to fluctuations in t-PA and PAI-1 levels, especially if those fluctuations are counter-balanced by other gender-related factors (i.e. other fibrinolysis inhibitors, hormones).

### Comparison of the GFC and the ECLT assays

GFC and ECLT are both coagulolytic assays but belongs to two different kinds. Table 2 summarizes the methodological differences between the two assays.

**Table 2:** Methodological comparison of ECLT and GFC

	Euglobulin clot lysis time	Global fibrinolysis capacity
Preanalytical	Binding, with sample collection, centrifugation and processing on melting ice to keep it at 4 °C	Sample collection, centrifugation and processing at room temperature
Measurement	Plasma sample artificially depleted in fibrinolysis inhibitors to mainly keep the euglobulin fraction (mainly fibrinogen, plasminogen and plasminogen activators (t-PA and u-PA))	‘Native’ plasma with fibrinolysis activators and inhibitors
Clot formation	Triggered by addition of CaCl_2_ → Endogenous thrombin → Endogenous fibrinogen	Triggered by addition of CaCl_2_ and exogenous human thrombin in large amount → Exogenous human thrombin → Endogenous fibrinogen
Clot lysis	Natural	Induced by the addition of micronized silica and recombinant t-PA (100 ng/mL)
Locally established reference range	131 – 523 min	29.3 – 49.5 min

First, the procedure we use to perform ECLT assay does not involve addition of exogenous thrombin in contrast to GFC assay. Thrombin is a key enzyme in coagulation, with both procoagulant (activation of factors V, VIII and XI, conversion of soluble fibrinogen into fibrin clot, activation of factor XIII) and anticoagulant effect (activation of protein C). For three decades now, thrombin is also known as playing a pivotal role between coagulation and fibrinolysis by converting TAFI into activated TAFI (TAFIa), a carboxypeptidase acting as a potent attenuator of fibrinolysis by counteracting the conversion of plasminogen into plasmin [47]. An addition of exogenous human thrombin, as is the case in the GFC assay, could therefore hide a defective activation of TAFI by the patient's endogenous thrombin, leading to a falsely normal or prolonged clot lysis time.

Second, to let endogenous clot lysis occur in ECLT, plasma samples are largely and on purpose depleted in fibrinolysis inhibitors; thus the assay is intended to detect hyperfibrinolysis due to an excess in circulating, uninhibited activators. In contrast, GFC is performed on ‘native’ plasma samples with addition of a low amount of recombinant t-PA and silica (activation of endogenous plasma fibrinolysis through the contact phase) to trigger the clot lysis, leading to the risk of potentially rendering the assay insensitive to the action of endogenous plasminogen activators. GFC seems then rather intended to explore decreased responsiveness to exogenously activated fibrinolysis. Nevertheless, GFC has shown some potential in the detection of hyperfibrinolysis in the context of orthotopic liver transplantation [14, 15] and trauma [16] as an attempt to identify patients who would benefit from tranexamic acid administration the most.

Third, local ECLT and GFC reference intervals were not established at the same time nor on the same healthy volunteers. If we look closely at Fig. 7, we can see that the points located in the C2 area are very close to those located in the B2 area (concordance of the results between the two methods). It is possible that the difference observed is rather due to a reference interval too stringent for the ECLT (the values being very close to the lower limit) rather than to a true disagreement between the two assays.

### Limitations of the study

This study displays some limitations. First, it was a monocentre study with a relatively small number of patients included (*n* = 43), and no case of acute hyperfibrinolysis. Second, reference interval has been determined with a relatively small number of healthy volunteers (*n* = 22). Third, individual determination of the major components of the fibrinolysis system have not been performed yet, but it is planned to investigate the discrepancies observed between men and women (for ECLT), and between ECLT and GFC results. Fourth, our procedure to perform ECLT assay does not involve the addition of exogenous thrombin in contrast to GFC assay. A further study comparing GFC and ECLT in the presence of exogenous thrombin could address whether the discrepancies we have observed between both assays could result from a defect in TAFI activation by thrombin. Finally, GFC reagent 1 (R1) contains silica in unspecified concentrations, which is known to activate not only coagulation but also fibrinolysis through the contact system [48]. As this reagent contains both silica and r-tPA, we did not assess the contribution of the contact phase to the determination of the GFC of plasma.

## Conclusion

GFC showed performance consistent with the manufacturer's claims and allows laboratories to perform analysis for non-urgent samples in batch with fresh reagents in one hand, and on the other hand to immediately analyze urgent samples using aliquoted frozen and thawed reagents. While the processing temperature did not have any influence on the GFC, a freeze-thawing cycle leads to a statistically significant, albeit slight, increase in the GFC. Centrifugation scheme and subsequent residual platelet count had no influence on plasma PAI-1 activity level, GFC or ECLT. Further studies regarding the influence of several freeze-thawing cycles on GFC are worth to be carried out, as well as studies to investigate the differences observed between fresh and frozen plasma samples. In summary, the GFC assay, which displays fewer pre-analytical constraints compared to ECLT, is suitable and convenient for a broad clinical use and can be performed with frozen-thawed plasma samples. The analytical performance we found was consistent with manufacturer's.

To the best of our knowledge, we established for the first time a reference range in adults with fresh plasma and the latest version of the assay. It is in line with previously reported data with earlier versions of GFC. Whether the slight bias we evidenced attributable to the freeze-thawing cycle mandates the determination of the corresponding reference range remains to be studied.

By design, GFC can detect hypofibrinolytic states since it takes into account the inhibitors. Whether it is as suitable as or even better than ECLT to detect a bleeding tendency due to a hyperactive fibrinolysis system deserves to be properly investigated, especially in the IVDR EU 2017/746 era.

## Data Availability

Supporting data are available from the corresponding author on request.
